# Iron Chelator Deferoxamine Alleviates Progression of Diabetic Nephropathy by Relieving Inflammation and Fibrosis in Rats

**DOI:** 10.3390/biom13081266

**Published:** 2023-08-18

**Authors:** Yunfei Feng, Li Jia, Wan Ma, Chenying Tian, Huahua Du

**Affiliations:** 1 Department of Endocrinology and Metabolism, First Affiliated Hospital, School of Medicine, Zhejiang University, Hangzhou 310003, China; fyf_med@zju.edu.cn; 2 MoE Key Laboratory of Molecular Animal Nutrition, College of Animal Sciences, Zhejiang University, Hangzhou 310058, China

**Keywords:** iron-chelator, deferoxamine, DFO, diabetic nephropathy, fibrosis, inflammation

## Abstract

Diabetic nephropathy (DN) is one of the most devastating diabetic microvascular complications. It has previously been observed that iron metabolism levels are abnormal in diabetic patients. However, the mechanism by which iron metabolism levels affect DN is poorly understood. This study was designed to evaluate the role of iron-chelator deferoxamine (DFO) in the improvement of DN. Here, we established a DN rat model induced by diets high in carbohydrates and fat and streptozotocin (STZ) injection. Our data demonstrated that DFO treatment for three weeks greatly attenuated renal dysfunction as evidenced by decreased levels of urinary albumin, blood urea nitrogen, and serum creatinine, which were elevated in DN rats. Histopathological observations showed that DFO treatment improved the renal structures of DN rats and preserved podocyte integrity by preventing the decrease of transcripts of nephrin and podocin. In addition, DFO treatment reduced the overexpression of fibronectin 1, collagen I, IL-1β, NF-κB, and MCP-1 in DN rats, as well as inflammatory cell infiltrates and collagenous fibrosis. Taken together, our findings unveiled that iron chelation via DFO injection had a protective impact on DN by alleviating inflammation and fibrosis, and that it could be a potential therapeutic strategy for DN.

## 1. Introduction

Diabetic nephropathy (DN) is a prominent complication of diabetes mellitus, accounting for approximately half of all causes of renal failure [1]. Renal hypertrophy, accumulation of mesangial matrix, proliferation of mesangial cells, and eventually glomerulosclerosis are characteristics of DN [2]. Proteinuria is regarded as a defining feature of DN since it represents a significant manifestation of glomerular damage. The prevalence of proteinuria in patients with type 2 diabetes mellitus (T2DM) has been shown to range from 20 to 40% in the absence of treatment [3].

Abnormal iron metabolism levels are commonly observed in diabetic patients. To be specific, the serum iron level, serum ferritin level, and transferrin saturation of diabetic patients were higher than those of healthy individuals, while serum hepcidin levels were considerably lower [4]. It has been demonstrated that the expression of divalent metal transporter 1, which is vital for iron uptake in most cells, was significantly improved in the small intestine of diabetic patients [5]. Our previous results showed that different dietary iron levels had significant effects on liver glycogen deposition, blood glucose and insulin levels in *db/db* mice and revealed that dietary high iron levels could deteriorate the development of T2DM [6]. In contrast, glucose tolerance and insulin secretion in diabetic animals can be improved by lowering iron levels. Feeding T2DM mice with low-iron diets or oral iron-chelators led to significantly improved insulin sensitivity, glucose tolerance and β-cell function [7]. Iron-chelating compound M30 could improve insulin secretion and glucose tolerance in T2DM mice [8]. Overall, these studies indicate that iron levels can affect the development of T2DM, and iron-chelating compounds may serve as potential therapeutic drugs for the treatment of T2DM and its complications.

Deferoxamine (DFO), deferasirox, and deferiprone are the three main iron-chelating substances currently used. All three are extremely selective for iron and have little or no impact on levels of calcium, lead, copper, phosphate, or magnesium [9]. DFO requires parenteral administration, whereas deferasirox and deferiprone can be taken orally. Traditional monotherapy with oral chelators, deferasirox, and deferiprone may not always achieve optimal control of iron overload in all patients [10]. DFO, an FDA-approved iron-chelating agent, was originally utilized in clinical practice during the 1960s and is frequently employed as a first-line treatment for individuals with iron-overload disease, such as hemochromatosis [11]. The primary mechanisms through which DFO exerts its neuroprotective effects are the chelation of a tiny portion of unbound iron, antioxidation, and elevation of hypoxia-inducible factor (HIF)-1 protein levels, which in turn modulate gene expression [12]. DFO has been shown to reduce hepatic lipid peroxidation and oxidative stress in rats [13] and ameliorate hepatic steatosis in *ob/ob* mice [14]. Moreover, DFO has been demonstrated to enhance insulin receptor activity and signaling in hepatocytes both in vitro and in vivo, as well as glucose uptake [15]. However, it is unclear if DFO can eliminate extra iron from particular kidney regions impacted by DN, and its precise mechanisms of action are still unknown.

In the current study, DFO was intraperitoneally injected into STZ-injected DN model rats to investigate a potential function of iron on DN. We discovered that the decrease of iron levels by DFO improved the progression of DN by reducing proteinuria and preserving ultrastructures of glomerulus and renal tubules through the inhibition of inflammation and fibrosis in the kidney.

## 2. Materials and Methods

### 2.1. Experimental Animals and Design

**Figure 1:**
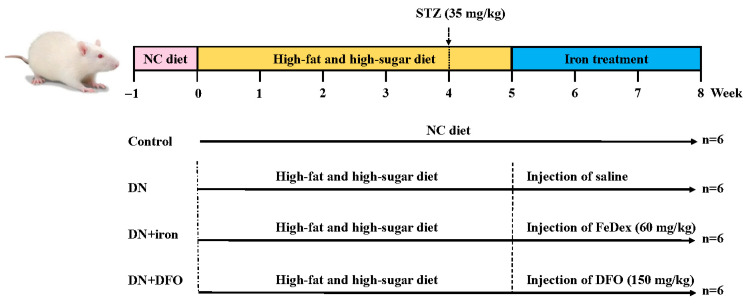
Outline scheme of experimental designs.

Male Wistar rats (seven-weeks-old) were procured from Zhejiang Chinese Medical University Laboratory Animal Research Center. They were housed in standard laboratory conditions (24 ± 2 °C) with food and water *ad libitum* on an alternate 12-h light/dark cycle. Prior to the commencement of the experiment, the rats were acclimatized to laboratory conditions for 7 d and fed a normal chow (NC) diet (Keao Xieli Feed, China; http://www.keaoxieli.com/product/136.html, accessed on 1 September 2019). Six rats were assigned to the control group, while the other 18 rats were used to establish a DN model (Figure 1). DN model rats were fed for 4 weeks with high-fat and high-sugar diets (10.0% lard, 20.0% sucrose, 2.5% cholesterol, 10% cholate, 66.5% conventional feed) and then injected intraperitoneally with 35 mg/kg b.w. of streptozotocin (STZ, Sigma-Aldrich, St. Louis, MI, USA) according to previous research [16]. The control rats were given an equivalent volume of sodium citrate buffer after 4 weeks of receiving the NC diet. Urine samples were taken each 24 h, and the urinary protein concentration was measured using an immunoassay (DCA 2000 System, Bayer Diagnostics, Elkhart, USA). Non-fasting blood glucose levels over 300 mg/dL (16.7 mmol/L) and urinary protein levels over 30 mg/24 h indicate the successful establishment of the DN model [1]. The DN model rats were separated into three additional groups: an untreated group (DN group), injection intraperitoneally with 60 mg Fe/kg b.w. of iron dextran (Fedex, Pharmacosmos A/S, Holbaek, Denmark) every two days for a week (DN + iron group), and injection intraperitoneally with 150 mg/kg b.w. of deferoxamine (DFO, Sigma-Aldrich, St. Louis, USA) once a day for a week (DN + DFO group) according to previous research [14]. Body weight and random blood glucose were detected weekly. All rats were sacrificed by cervical dislocation at 8 weeks after the treatment. All the experimental protocols were approved by the Animal Ethics Committee of Zhejiang University.

### 2.2. Serum and Urine Biochemical Assays

A week after STZ injection, blood samples were collected from the tail vein under anesthesia and subsequently serum glucose concentrations were evaluated using a Contour TS Meter Glucometer (Bayer Diagnostics, Leverkusen, Germany). Serum transferrin level was measured using an ELISA kit (Huamei Biology, Wuhan, China). At the end of the experiment, the rats were sedated, and blood was drawn from their hearts for further measurements. The levels of serum triglycerides, cholesterol, and low-density lipoprotein (LDL) were measured using ELISA kits (MBbiology, Nanjing, China) according to the manufacturer’s recommendations. Urine albumin and creatinine levels were measured by using Albuwell M kit and the Creatinine Companion kit (Exocell, Philadelphia, PA, USA), respectively, based on the manufacturer’s instructions.

### 2.3. Histological Analysis of Renal Tissues

The kidneys were taken out and fixed in 4% paraformaldehyde at 4 °C for 24 h. Hematoxylin-eosin (H&E), periodic acid-Schiff (PAS), and Masson-trichrome staining were employed to analyze the histology changes by using 3-μm sections [17]. For iron staining, paraffin-embedded kidney samples were cut into 2-μm slices before being stained with Prussian blue. At the same time, slices were heated to facilitate the removal of antigens in citrate buffer, followed by overnight incubation at 4 °C with primary antibodies against ferritin heavy chain (FtH, 1:200; Uscn Life Science, Wuhan, China) as described previously [18]. For transmission electron microscope (TEM) analysis, kidney samples were fixed in cacodylate buffer containing 2.5% glutaraldehyde, dehydrated using graded alcohol (50, 70, 90, and 100%), and transferred to pure acetone. The specimens were submerged in acetone and Spurr resin (1:1 for 1 h, 1:3 for 3 h), and then moved to Spurr resin mixture overnight. Ultrathin sections were cut into 70 nm and stained with uranyl acetate and alkaline lead citrate and observed under a TEM (H-7650, Hitachi, Japan).

### 2.4. Quantitative PCR Analysis

Using the Prime Script RT kit, cDNA was produced after total RNA was extracted using the TRIzol reagent (Takara, Shiga, Japan). Real-time PCR was carried out using the ABI 7500 Real-time PCR System (Applied Biosystems, Waltham, MA, USA) and FastStart Universal SYBR Green Master (Roche, Boston, MA, USA). Using β-actin mRNA as a control, the 2^−ΔΔ^ cycle threshold approach was used to calculate the mRNA expression level. A melting curve analysis was used to monitor specificity and each reaction was run at least three times. The specific primers’ sequences are provided in Table 1.

### 2.5. Statistical Analysis

All assays were performed at least three times and the data were expressed as the mean ± standard error of the mean (SEM). The statistical analysis was performed using GraphPad Prism version 8.0 (GraphPad Software, Boston, MA, USA). The differences between the two groups were compared using the unpaired, two-tailed Student’s *t* test. The significance level was set at *p* < 0.05. Differences that are statistically significant are shown by the symbols * *p* < 0.05 and ** *p* < 0.01.

## 3. Results

### 3.1. Effects of DFO on the Body Weight and Biochemical Parameters

**Figure 2:**
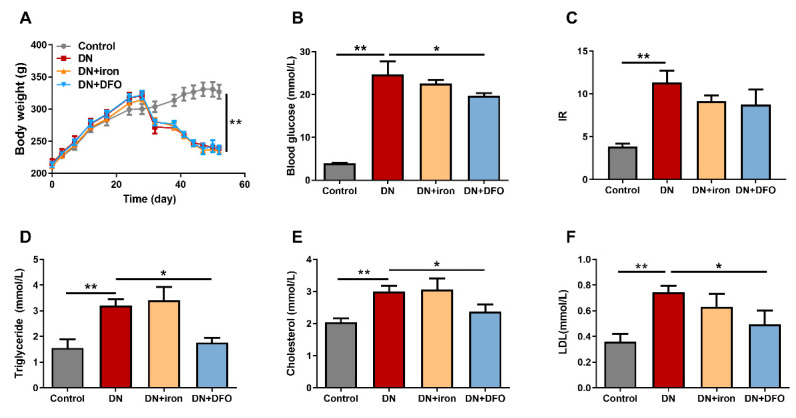
Effects of DFO on the body weight and biochemical parameters. The curve graph showed the body weight changes of rats (**A**). The bar graphs summarized average values for blood glucose levels (**B**), IR (**C**), plasma triglyceride levels (**D**), plasma cholesterol concentrations (**E**), and LDL concentrations (**F**). IR: insulin resistance; LDL: low-density lipoprotein. Values were expressed as the mean ± SEM, *n* = 6 in each group. Asterisks indicate a significant difference (* *p* < 0.05, ** *p* < 0.01).

A DN rat model was established successfully (non-fasting blood glucose 24.78 ± 3.0 mmol/L, insulin-resistance index 11.35 ± 1.36, and urine protein 45.34 ± 1.15 mg/24 h) according to the criteria for DN [1]. The body weight of rats was significantly (*p* < 0.01) decreased in the DN groups (Figure 2A). Treatment with iron and DFO had no effect on body weight change. However, there were significant increases in the levels of blood glucose, IR, triglyceride, cholesterol, and LDL in the DN group compared with the control group (Figure 2B–F). The increase of blood glucose, triglyceride, cholesterol, and LDL were significantly (*p* < 0.05) inhibited by administration of DFO (Figure 2B,D–F). No significant difference was observed between the DN group and the DFO-treated DN groups for IR levels (Figure 2C). These findings suggest that DFO protects DN rats from metabolic impairment.

### 3.2. Effects of DFO on Iron Levels in Diabetic Kidneys

**Figure 3:**
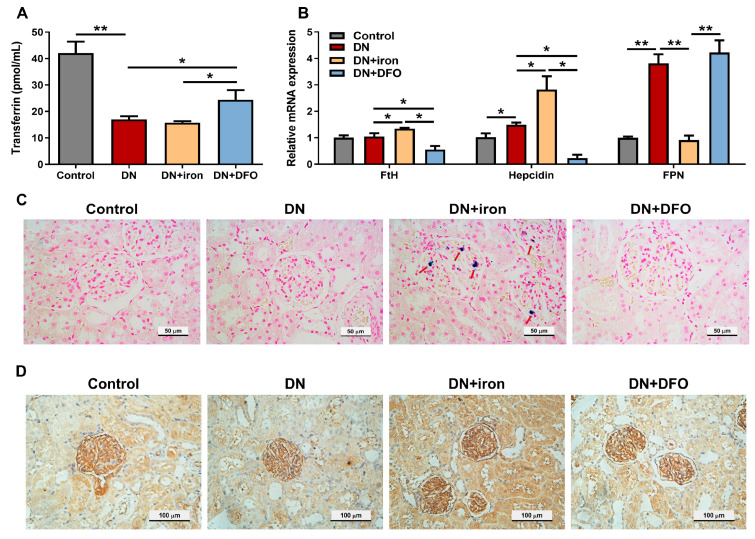
Effects of DFO on iron levels in diabetic kidneys. (**A**) The concentration of transferrin in serum. (**B**) Relative mRNA expression of FtH, hepcidin, and FPN normalized to β-actin was examined by qRT-PCR. (**C**) Non-heme iron deposits in the kidney were detected by staining with Perls’ Prussian blue and the cells counterstained with nuclear red. (**D**) Representative immunohistochemical staining of FtH in the kidney. FtH: ferritin heavy chain; FPN: ferroportin. Values were expressed as the mean ± SEM, *n* = 6 in each group. Asterisks indicate a significant difference (* *p* < 0.05, ** *p* < 0.01).

Serum transferrin concentration was significantly (*p* < 0.01) decreased in DN groups (Figure 3A). Treatment with DFO increased the transferrin level of DN rats significantly (*p* < 0.05), while treatment with iron had no effect. Compared with DN rats, the mRNA expression of renal FtH and hepcidin was significantly (*p* < 0.05) increased in DN + iron rats, but significantly (*p* < 0.05) decreased in DFO-treated DN rats (Figure 3B). However, renal FPN expression was decreased significantly (*p* < 0.01) in DN + iron rats and restored in DN + DFO rats compared to DN rats. In addition, increased iron deposits were discovered in the kidneys of rats receiving iron supplementation, as opposed to none in the kidneys of other rats, according to a Prussian blue staining assay (Figure 3C). As shown in Figure 3D, FtH mRNA expression was augmented in the kidneys of DN + iron rats, although there was no significant difference between DN rats and DN + DFO rats.

### 3.3. Effects of DFO on Renal Function in DN Rats

**Figure 4:**
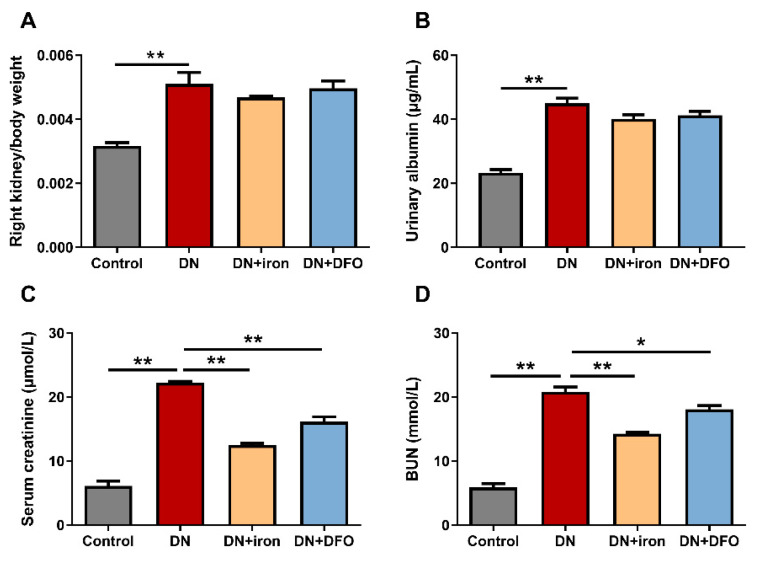
Effect of DFO on renal function in DN rats. Right kidney weight (**A**) and urinary albumin levels (**B**) were not altered by iron or DFO treatment. The serum creatinine (**C**) and BUN levels (**D**) were decreased by iron or DFO treatment. BUN: blood urea nitrogen. Values were expressed as the mean ± SEM, *n* = 6 in each group. Asterisks indicate a significant difference (* *p* < 0.05, ** *p* < 0.01).

We then narrowed our attention to kidney function and assessed the potential anti-diabetic effects of DFO. When exposed to STZ, the ratio of right kidney weight to body weight was increased, while iron or DFO had no effect on this tendency (Figure 4A). The considerable renal impairment caused by STZ treatment was demonstrated by elevated levels of urinary albumin, serum creatinine, and BUN (Figure 4B–D). Although DFO administration did not affect the level of urine albumin, it dramatically decreased the levels of serum creatinine (*p* < 0.01) and BUN (*p* < 0.05). Similar results to those shown in DFO-treated rats were seen when iron was treated. According to the aforementioned research, DFO did in fact have a preventive effect against DN.

### 3.4. Effects of DFO on Kidney Structures in DN Rats

**Figure 5:**
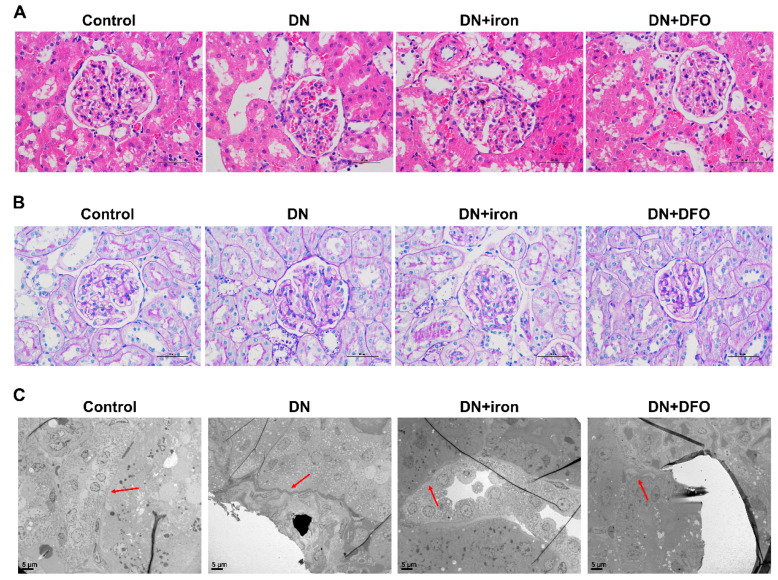
Effect of DFO on renal morphology in DN rats. (**A**) H&E staining. Representative micrographs were shown. Scale bar = 50 μm. (**B**) PAS staining. Representative micrographs were shown. Scale bar = 50 μm. (**C**) TEM analysis. Arrowheads indicate glomerular basement membrane. Scale bar = 5 μm. PAS: Periodic Acid-Schiff stain; TEM: transmission electron microscope.

We then examined the effect of DFO on the pathophysiology of DN. H&E staining revealed thickening of the basement membrane in the glomerular capillary and broadening of the mesangial area in DN rats, while these pathological changes were exacerbated by iron, but ameliorated by DFO treatment (Figure 5A). PAS staining revealed that DN rats had a larger glomerular area and more extensive mesangial expansion compared with the control rats, which was stimulated by iron, but suppressed by DFO treatment (Figure 5B). TEM imaging further proved that glomerular capillary basement membrane thickening occurs in DN rats, with these pathological changes ameliorated by DFO treatment (Figure 5C).

### 3.5. Effects of DFO on Podocyte Foot in DN Rats

**Figure 6:**
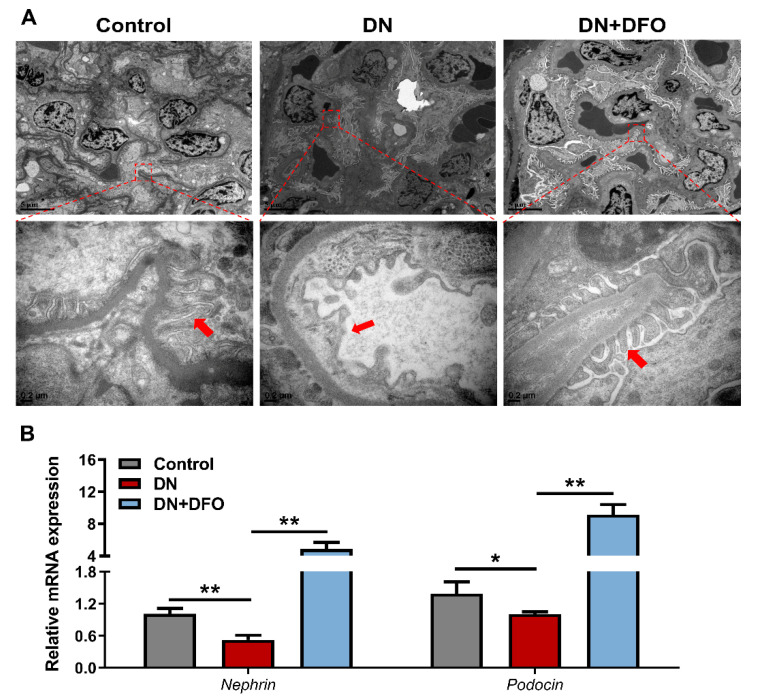
Effects of DFO on podocyte morphology in DN rats. (**A**) TEM analysis. Arrowheads indicate podocytes. Scale bar = 5 μm (upper). Scale bar = 0.2 μm (under). TEM: transmission electron microscope. (**B**) Relative mRNA expression of nephrin and podocin normalized to β-actin was examined by qRT-PCR. Values were expressed as the mean ± SEM, *n* = 6 in each group. Asterisks indicate a significant difference (* *p* < 0.05, ** *p* < 0.01).

Since iron treatment had a slight effect on DN rats, we focused our interest on DFO-treated animals in the following experiments. In order to better understand the aforementioned occurrences, we used TEM to investigate the ultra-structures of the podocyte foot process. The results revealed that DN rats experienced more severe foot process effacement, whereas DFO treatment largely reversed these detrimental consequences (Figure 6A). We then explored how DFO affected the gene expression of nephrin and podocin in DN because STZ can cause podocyte injury by decreasing nephrin and podocin expression [19]. As compared with the control rats, the mRNA expression of both nephrin (*p* < 0.01) and podocin (*p* < 0.05) were substantially downregulated in DN rats. As expected, DFO treatment dramatically stopped the declines of transcripts of nephrin and podocin (Figure 6B). These data clearly indicated that DFO preserved podocyte integrity in DN rats and played a beneficial role against DN.

### 3.6. Effects of DFO on Kidney Fibrosis in DN Rats

**Figure 7:**
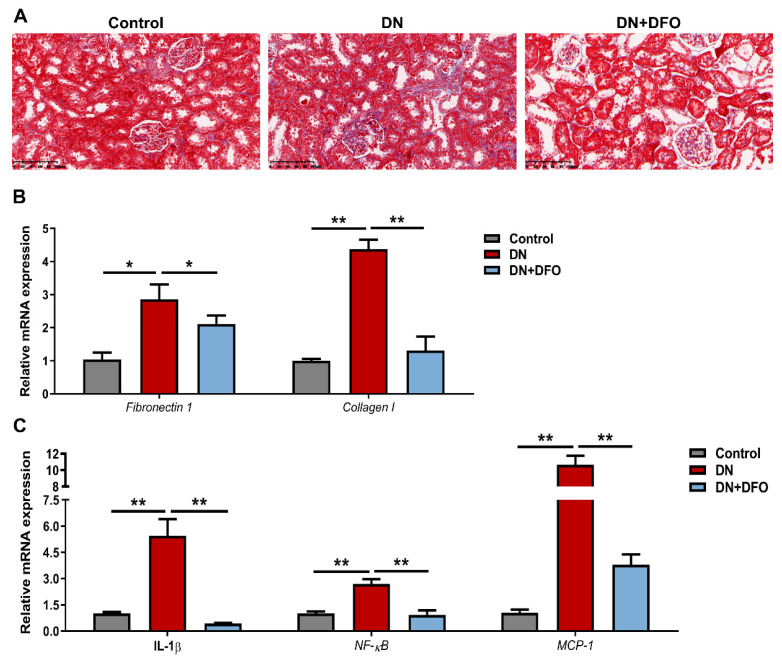
Effects of DFO on kidney fibrosis in DN rats. (**A**) Masson staining. Representative micrographs were shown. Scale bar = 100 μm. (**B**) Relative mRNA expression of fibronectin 1 and collagen I normalized to β-actin was examined by qRT-PCR. (**C**) Relative mRNA expression of IL-1β, NF-κB, and MCP-1 normalized to β-actin was examined by qRT-PCR. Values were expressed as the mean ± SEM, *n* = 6 in each group. Asterisks indicate a significant difference (* *p* < 0.05, ** *p* < 0.01).

DN is characterized by tubulointerstitial fibrosis and atrophy [2]. We then studied the impact of DFO on kidney histology using Masson-trichrome staining and examined the mRNA levels of myofibroblast markers to determine whether DFO reduced renal fibrosis. Masson staining showed a significant increase in inflammatory cell infiltrates in the interstitium and collagenous fibrosis in DN rats as compared to control rats, while DFO distinctly reversed these alterations (Figure 7A). In DN rats, the mRNA expression of fibronectin 1 (*p* < 0.05) and collagen I (*p* < 0.01) was dramatically enhanced, and DFO treatment resulted in a striking reduction (Figure 7B). The mRNA expression of pro-inflammatory cytokines including IL-1β, NF-κB, and MCP-1 showed a similar trend (Figure 7C).

## 4. Discussion

The findings of the current study showed that DFO could slow the advancement of albuminuria, mesangial area expansion, extracellular matrix deposition, and renal podocyte injury in diabetic rats induced by STZ injection. These results suggested that iron restriction might have a protective effect against the development of DN. Our study also demonstrated that DFO treatment decreased collagenous fibrosis and inflammatory cell infiltrates in DN rats. As a result, the reduction of inflammation and fibrosis is a contributing factor in iron restriction’s protective effect against DN.

Epidemiologically, higher iron status has been positively associated with the risk of T2DM [20]. Although the pathogenic mechanism remains unknown, the detrimental effects of iron overload on glycemic regulation may already be reflected in numerous tissues. Firstly, higher iron levels have the potential to impair the function of pancreatic β cells in both people and animals fed a high-fat diet [21]. Secondly, mice fed a high-iron diet with elevated iron status may have reduced insulin sensitivity and adiponectin secretion in adipocytes [22]. Thirdly, excess intake of dietary iron has been found to increase the activity of adenosine monophosphate-activated protein kinase C in the liver and skeletal muscle and impair insulin signaling pathways in mice [23]. Finally, the enzyme heme oxygenase-1 may have increased long-term metabolic inflammation and insulin resistance in macrophages and hepatocytes by decomposing heme into carbon monoxide, biliverdin, and free iron [24]. The risk of T2DM is associated with a number of iron indices, including ferritin, transferrin saturation, and heme iron consumption, according to multiple meta-analyses [25]. Genetically instrumented serum iron, ferritin, and transferrin saturation were all positively correlated with T2DM, while transferrin, a marker of low iron status, was negatively correlated with T2DM [26]. Our previous studies have shown that a high-iron diet could deteriorate the development of T2DM in diabetic mice [6]. On the other hand, chelation therapy or iron reduction by phlebotomy lessened the effects of diabetes. Experimental animal trials exploring dietary iron reduction, phlebotomy, and iron chelation therapy all revealed similar positive outcomes [27]. Despite the fact that systemic distribution of DFO does not provide diabetic patients a feasible therapeutic alternative because of probable toxicity and a brief plasma half-life, local transdermal drug delivery systems containing DFO have been found to be highly effective for the prevention of pressure-induced diabetic ulcers [28]. In our study, DFO treatment significantly attenuated renal dysfunction, as shown by lower levels of urinary albumin, blood urea nitrogen, and serum creatinine, all of which were elevated in DN rats. DFO chelates ferric iron to produce stable complexes that keep iron out of the Fenton processes. After systemic injection, DFO is rapidly absorbed and disseminated throughout all body fluids. Most frequently, oxidative deamination is used to metabolize DFO, resulting in metabolite B, which is quickly eliminated by the kidneys with its iron-chelating complex [29]. As expected, our study demonstrated that treatment with DFO increased the transferrin level of DN rats, while significantly reducing the expression of renal FtH.

Podocytes are the primary focus of many renal illnesses and play an important role in the maintenance of normal kidney function. The glomerular filtration barrier is supported by podocytes, which have a unique slit diaphragm [30]. Podocytes play an essential role in building and maintaining the filtration process of the glomerulus. Podocytes have four basic functions: the regulation of glomerular permeability selectivity, provision of structural support for the glomerular capillary, remodeling of the glomerular basement membrane, and endocytosis of filtered proteins [31,32]. It is now recognized that DN is characterized by podocyte damage and dysfunction [33]. Foot processes are composed of several proteins, of which nephrin is the earliest identified and most studied. Nephrin is an essential component of podocytes which combines with endothelial cells and the basement membrane to form the glomerular filtration barrier [34]. Podocyte damage results in nephrin release. Nephrin can be detected in the urine as the result of podocytopathies. Urine nephrin analysis has become an important biomarker of early glomerular urinary injury and is an earlier, more sensitive, and specific marker of DN than microalbuminuria [35,36]. The expression of nephrin protein is reduced both in humans and rats with DN. Another vital protein called podocin is exclusively expressed in podocytes of developing and mature glomeruli and is located in the cytosolic side of the slit diaphragm [37]. It acts together with other transmembrane adhesion proteins such as nephrin to form a protein complex. Importantly, podocin interaction is required for effective signaling through nephrin and its associated proteins [38]. Examining urinary levels of podocin helps to evaluate podocyte loss and monitor treatment response of DN [39]. In our experiment, we found that severe effacement of foot process occurred, and gene expression of nephrin and podocin were both substantially down-regulated in DN rats. Such a situation was reversed after DFO treatment. DFO remarkably prevented the decreases of transcripts of nephrin and podocin, and noticeably improved the ultra-structures of the podocyte foot process. This finding is consistent with previous reports that mesenchymal stem cell-derived conditioned media pre-incubated with DFO could be more effective in the treatment of DN by reducing podocyte damage and tubular apoptotic cell death [40].

One major pathological alteration of advancing DN is renal fibrosis, which promotes severe disruption of kidney structure and function. The degree of renal cortical interstitial fibrosis and serum creatinine levels at the time of biopsy are positively correlated in patients with DN [41]. The Fenton reaction, which can be fueled by iron overload, can produce enormous numbers of free radicals that cause substantial damage to cells and tissues and induce fibrosis [42]. Moreover, excess iron might cause fibrosis-promoting signals, which quicken the onset of illness and aggravate kidney pathology. Coincidentally, our experiments confirmed that a significant increase in inflammatory cell infiltrations in the interstitium and collagenous fibrosis were observed in DN compared with control rats, and these pathological changes were obviously alleviated by treatment with DFO. In addition, progressive kidney fibrosis is caused by profibrotic cytokines and growth factors, which are produced and released as a consequence of chronic renal inflammation [43]. Kidney damage in diabetes is caused by the secretion of pro-inflammatory chemicals by inflammatory cells. In our study, we observed a significant increase in the mRNA expression of IL-1β, NF-κB, and MCP-1 in renal tissues, pointing to an inflammatory response in the kidneys of diabetic rats. It’s interesting to note that DFO therapy reduced the expression of these cytokines.

**Figure 8:**
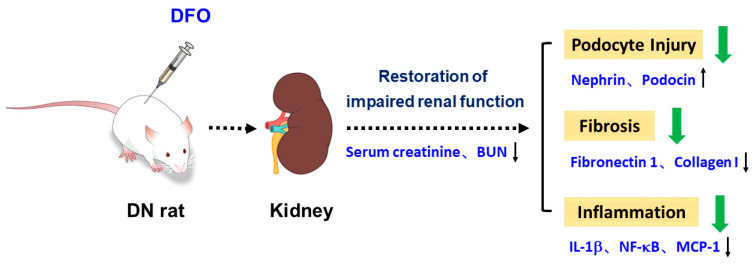
DFO protects against DN via attenuating podocyte injury, fibrosis, and inflammation in STZ-induced rats.

In summary, our finding indicated that DFO has a renoprotective impact on the progression of DN, and its potential mechanism may be related to the prevention of inflammation, fibrosis, and podocyte injury (Figure 8). The ideal anti-diabetic dose of DFO will be determined through dose-dependent research. Long-term administration of DFO to rats with normal renal function and those with renal failure is required to prove its efficacy and safety in the future. Furthermore, while most studies used male animals to establish DN models [17,19,28], further research into sexual dimorphism in DN is needed not only to advance our overall understanding, but also to develop DFO therapy that takes into consideration the inherent differences in pathophysiology between the sexes.

## Figures and Tables

**Table 1:** Primer Sequences for the real-time PCR amplification.

Gene	Forward Primers	Reverse Primers
β-actin	CGCCAACCGCGAGAAGAT	CGTCACCGGAGTCCATCA
FtH	TCAGTCACTACTGGAACTGC	CGTGGTCACCCAGTTCTTTA
Hepcidin	TTGCGATACCAATGCAGAAG	TGCAACAGATACCACACTGG
FPN	GAATAATGGGAACTGTGG	AAGTGGCTCTGTCTGAAT
Nephrin	GACACGAGAAGCTCCACGGTTA	GTCGTAGATTCCCCTCGGATC
Podocin	GCCTCCCTTCTTCTAAGCAGTCTA	TCAGTTCTCTCCACTTTGATGCC
Fibronectin 1	ACAGAGCTCAACCTCCCTGA	TGTGCTCCTGGTTCTCCT
Collagen I	TCACCACAATGCCGTTC	GCCACTAATTGGAGCCATGT
IL-1β	ACAAAAGCCCGTCTTCCTG	ATGTGGACCTCTGGGTATGG
NF-κB	AAGCACTGCAGGGAGACTGT	ATCTTGAGCTCGGCAGTGTT
MCP-1	CAAGAGAATCACCAGCAGCA	AAGCTCATGCAAATGGAAGG

## Data Availability

The analyzed data sets generated during the present study are available from the corresponding author upon reasonable request.
